# Opioid Use and Family Members' Experiences of Opioids in Paediatric Cancer Pain Management: An Integrative Review

**DOI:** 10.1111/scs.70323

**Published:** 2026-09-30

**Authors:** Noora Puusa, Eeva Harju, Maija‐Liisa Kalliomäki, Elina Haavisto

**Affiliations:** ^1^ Faculty of Social Sciences, Unit of Health Sciences, Nursing Science Tampere University Tampere Finland; ^2^ Department of Gastroenterology and Alimentary Tract Surgery TAYS Cancer Center, Tampere University Hospital, Wellbeing Services County of Pirkanmaa Tampere Finland; ^3^ Department of Surgery, TAYS Cancer Center Tampere University Hospital, Wellbeing Services County of Pirkanmaa Tampere Finland; ^4^ Department of Anaesthesia Tampere University Hospital, Wellbeing Services County of Pirkanmaa Tampere Finland; ^5^ Faculty of Medicine and Health Technology, University of Tampere Tampere Finland; ^6^ Tampere University Hospital, Wellbeing Services County of Pirkanmaa Tampere Finland

**Keywords:** analgesia, analgesics, cancer, child, family, integrative review, neoplasm, opioid

## Abstract

**Aim:**

To analyse the use of opioids in children with cancer and the experiences of their family members regarding opioids used in pain management to improve family nursing.

**Design:**

An integrative review.

**Methods:**

A systematic literature search was carried out in four databases (CINAHL, MEDLINE, PsycInfo and Scopus) in August 2023, and was subsequently updated to include studies up to the end of 2024. Sixteen peer‐reviewed research articles met the inclusion criteria and were included in a quality appraisal and analysis.

**Results:**

Of the wide range of opioids used to treat pain in children with cancer, morphine and fentanyl stood out as the most used. The prevalent routes of opioid administration was intravenous and enteral. Limited research has been conducted on the experiences of family members of children with cancer in the context of opioids used for pain management. Furthermore, there is a scarcity of studies that describe the experiences of siblings from the same perspective. Parental experiences were particularly described as concerns about and a desire to refuse opioid use, although satisfaction with opioid use was also described.

**Conclusions:**

Opioids are particularly significant in managing pain among children with cancer. Therefore, the use of opioids in this patient group should be comprehensively studied, and healthcare professionals should receive sufficient training. The experiences of parents conclude that they require sufficient information and guidance on the management of their children's severe pain and the analgesics used to treat it. The notable lack of research regarding the experiences of siblings in connection with the use of opioids for the management of a child's pain addresses the need for further research from these perspectives. More research on experiences during the different phases of cancer treatment is needed. Further research employing qualitative methods will be essential for enhancing family nursing.

## 1 Introduction

Childhood cancers have a widespread impact on families globally, with approximately 400,000 children being diagnosed with cancer annually [1]. The most common childhood cancers are leukaemia, lymphoma and central nervous system tumours, although the prevalence of these cancers varies between different age groups. Leukaemia stands out as the predominant form of cancer among children aged between 0 and 4 years, whereas lymphoma is more common in children aged 15 to 19 years. In children, brain tumours are equally common in all age groups, but epithelial tumours and melanoma are more frequently diagnosed in older children and adolescent populations [1, 2]. The treatment of cancer in children causes significant changes in normal daily life, including adhering to a strict treatment protocol, undergoing various procedures, living with a central venous catheter and administering medications [1, 3, 4, 5].

Cancer and its treatments are associated with pain, which varies in intensity during different phases of the disease. As one of the most oppressive symptoms of cancer, pain in children can manifest as either acute, during or after treatment, procedural or prolonged, displaying variations in both intensity and location. This variability significantly compromises the overall quality of life of the child [5, 6, 7, 8]. The most common cause of pain is cancer and tumours [9], but the inflammation of the oral mucosa following treatment, referred to as mucositis, also emerges as a significant cause of pain in paediatric cancer patients [10].

The goal of pain management is to effectively alleviate and relieve pain at the earliest stage possible [1]. The administration of analgesics should be based on the evaluation of the pain level. Also, it is important to measure the effect of the analgesic by repeating the pain measurement, to adjust the dosing. To be able to achieve this goal, the caregivers should understand the level of pain of the child. As children of all ages and different stages of their cancer suffer from pain, the pain measurement method should be adjusted to the child's cognition stage. In infants, or children not able to express themselves, observing faces, legs, arms, cry and consolability (FLACC) is the best measurement [11, 12, 13, 14]. Wong‐FACES pain scale [15] is suitable for children three years and older and is based on a visual method where the child can point at a picture of a facial expression corresponding to their level of pain. Teenagers and older are able to express their pain by numbers (0–100, numerical rating scale) [16] or by the visual analog scale (VAS) [17]. Observing the behaviour, movement and sleeping of the child is also valuable to understanding the impact of pain on daily wellbeing. In the treatment of severe pain, the administration of strong analgesics such as opioids is often necessary. In addition to effective pain management, the use of opioids is also linked to various adverse effects, concerns and challenges [18]. Respiratory depression stands out as the most serious adverse effect of opioid use, with vomiting and itching being the most frequently reported [19]. Along with adverse effects, the use of opioids is intertwined with diverse cultural considerations and regional challenges caused by opioids [20]. Furthermore, the worldwide availability of opioids varies, leading to diverse access to comparable pain medications for individuals worldwide [21].

Although children with cancer are treated centrally in children's wards, treatment is nowadays increasingly provided in outpatient care or at home. Children in a palliative phase especially are often cared for in the familiar environment of their own home [22, 23]. The transition from traditional care settings to home‐based care represents an improvement in support for children with cancer, fostering better adaptation and improved quality of life [22, 24]. Nonetheless, this transition also entails the transfer of responsibilities and burdens to the family, thereby placing a significant load on the parents [22]. While most paediatric cancer patients survive, sadly not all patients do, leading to the need for palliative care. In palliative care, a strong focus is placed on the effective pain management of the child and the pivotal role of family members in ensuring the quality of the time the child has left [25, 26].

For a family, the serious illness of a child can cause a change in both routines and roles, shaping a new normal in the lives of family members [27]. Furthermore, cancer can also change the relationship between children and their siblings and between siblings and their parents, changing the dynamics of the whole family [28]. From the perspective of parents, the most challenging symptoms in the treatment of a child with cancer are anxiety, fatigue, nutrition and pain. Among these, the pain experienced by the child is the most challenging [29]. Additionally, parents not only have to contend with the worry and sadness of having a sick child but also must manage the emotions of their other children, while at the same time navigating existential questions about the cause of the illness and adjusting to a new normal in everyday life [27]. The occurrence of cancer in a child has a substantial impact on siblings, who have been identified as being at psychosocial risk and in need of adequate support services [28].

Previous studies have explored the effects of childhood cancer on the family [27] and reported positive connections between a child's pain levels and parental presence [30]. Although pain has been the focus of research for a remarkable period, there is a lack of research on the different pain management experiences of family members, particularly in cases of paediatric cancer patients who are using opioids. Therefore, to obtain a comprehensive description of the subject and to guide future research, an extensive examination of the current literature was undertaken through an integrative review. The review aims to enhance understanding and improve family nursing in the treatment of children with cancer and the management of pain in paediatric cancer patients.

The aim of this review was to analyse the use of opioids in children with cancer and to ascertain the experiences of family members regarding opioids used in pain management. The research questions are as follows:

- How are opioids used in the pain management of children with cancer?
- What kind of experiences do family members of a child with cancer have regarding the use of opioids in managing the child's pain?

## 2 Methods

An integrative review design allows the inclusion of studies conducted using various methodologies, resulting in a more comprehensive overview of the research topic. Integrative reviews are also appropriate for phenomena that are not well known and for enhancing knowledge in nursing practice. This integrative review was performed by following a framework comprising five different stages. The five stages were as follows: (1) problem identification, (2) literature search, (3) data evaluation, (4) data analysis and (5) presentation of the results [31]. The Preferred Reporting Items for Systematic reviews and Meta‐Analyses (PRISMA) guidelines were followed to ensure the transparency and accurate reporting of the literature search [32].

### 2.1 Literature Search

The literature search was conducted in August 2023 in four databases: CINAHL, MEDLINE, PsycInfo and Scopus, without publication time limitations. The search was subsequently updated to include studies published through the end of 2024. The search terms were child, cancer, opioid and pain, along with the synonyms of these search terms. Database‐specific MeSH terms and Boolean operators were also used. For the literature search, assistance was received from an academic health science librarian. To be eligible for selection, the articles had to fulfil the following inclusion criteria: (1) studies that describe a child with cancer aged 0 to 18 years, (2) use of opioid for pain management during any cancer treatment phase, (3) studies that describe the experiences of family members of children with cancer, (4) peer‐reviewed original research articles and (5) articles written in English, Swedish or Finnish. Family members were defined as the child's parents and siblings. A manual search was done by viewing the reference lists of the included studies.

Article titles and abstracts were screened by the first author. Full texts were then screened by two authors; unclear parts were discussed and a consensus about the quality of the included articles was reached. In total, 6451 research articles were identified through the database search. After duplicate records were removed, 5028 records were left for title screening. Irrelevant articles were then removed, leaving 565 articles for abstract screening. After screening the abstracts, 70 articles were left for screening through full text. A total of 54 articles were excluded based on the exclusion criteria. Thus, the total number of articles included in this review was 16. A manual search by reviewing the reference lists of the included studies failed to identify any further articles for addition to the review Figure 1.

**FIGURE 1:**
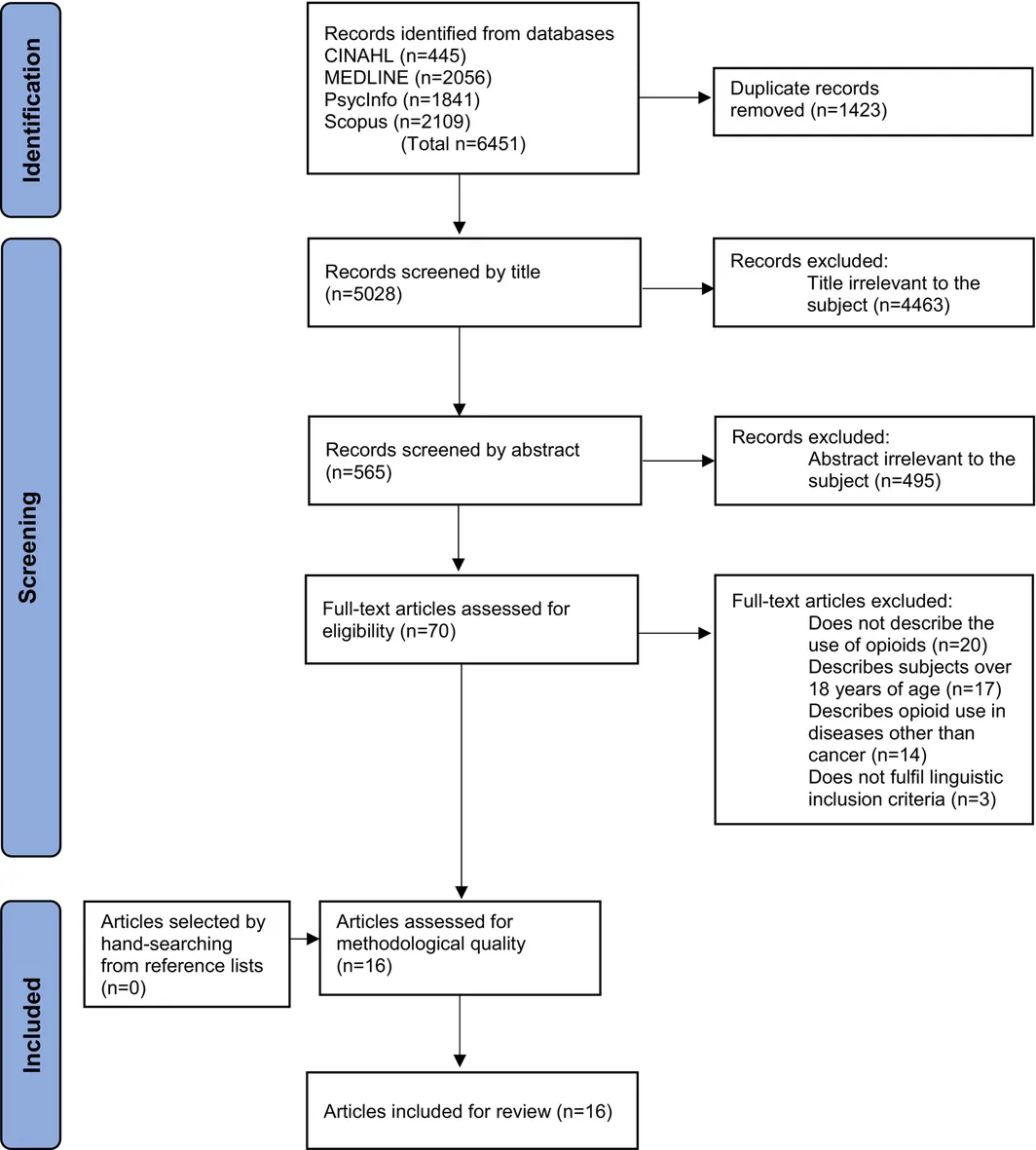
PRISMA flow diagram of the literature search [32].

### 2.2 Data Evaluation

Separate appraisal tools made by the Joanna Briggs Institute were used to evaluate the quality of the studies. The different study types and appraisal tools were cross‐sectional (8 questions), quasi‐experimental (9 questions), case reports (8 questions), case series (10 questions) and randomized controlled trials (13 questions) [33]. The quality appraisal of the articles was conducted independently by the first and second authors and the results were discussed and agreed upon on each criterion (Table 1). Most of the studies were rated as being of high quality. The lowest quality assessment scores were assigned to two case series (7/10 points), two cross‐sectional studies (5/8 points) and one case report (4/8 points). The main reasons that affected the quality scores related to ambiguous reporting about confounding factors in cross‐sectional studies. In case series, there was also a lack of valid methods used to identify the conditions of participants.

**TABLE 1:** Quality appraisal of the studies [33].

JBI appraisal checklist for analytical cross‐sectional studies	Kubica‐Cielinska et al. [34]	Drake et al. [35]	Yang et al. [36]	Sirkiä et al. [37]	JBI appraisal checklist for quasi‐experimental studies	Ruggiero et al. [38]	Mashayekhi et al. [39]	Othman et al. [40]
1 Were the criteria for inclusion in the sample clearly defined?	Yes	Unclear	Yes	Unclear	1 Is it clear in the study what is the ‘cause’ and what is the ‘effect’?	Yes	Yes	Yes
2 Were the study subjects and the setting described in detail?	Yes	Yes	Yes	Yes	2 Were the participants included in any comparisons similar?	Yes	Yes	Yes
3 Was the exposure measured in a valid and reliable way?	Yes	Yes	Yes	Yes	3 Were the participants included in any comparisons receiving similar treatment/care, other than the exposure or intervention of interest?	Yes	No	No
4 Were objective, standard criteria used for measurement of the condition?	Yes	Yes	Yes	Yes				
5 Were confounding factors identified?	Yes	Unclear	No	Unclear	4 Was there a control group?	No	No	No
6 Were strategies to deal with confounding factors stated?	Unclear	Unclear	No	Unclear	5 Were there multiple measurements of the outcome both pre and post the intervention/exposure?	Yes	Yes	Yes
7 Were the outcomes measured in a valid and reliable way?	Yes	Yes	Yes	Yes				
8 Was appropriate statistical analysis used?	Yes	Yes	Yes	Yes	6 Was follow up complete and if not, were differences between groups in terms of their follow up adequately described and analysed?	Yes	Yes	Yes
Total score	7/8	5/8	6/8	5/8				
					7 Were the outcomes of participants included in any comparisons measured in the same way?	Yes	Yes	Yes
					8 Were outcomes measured in a reliable way?	Yes	Yes	Yes
					9 Was appropriate statistical analysis used?	Yes	Yes	Yes
					Total score	8/9	7/9	7/9

JBI appraisal checklist for case series	Coombes et al. [41]	Davies et al. [42]	Noyes & Irving [43]	Anghelescu et al. [44]	Schiessl et al. [45]	Madden et al. [46]	JBI appraisal checklist for randomized controlled trials	Collins et al. [47]
Were there clear criteria for inclusion in the case series?	Yes	Yes	Yes	Yes	Yes	Yes	Was true randomization used for assignment of participants to treatment groups?	Yes
2 Was the condition measured in a standard, reliable way for all participants included in the case series?	Yes	Yes	Yes	Yes	Yes	Unclear	2 Was allocation to treatment groups concealed?	Yes
3 Were valid methods used for identification of the condition for all participants included in the case series?	Unclear	Unclear	Unclear	Yes	Yes	Unclear	3 Were treatment groups similar at the baseline?	Yes
4 Did the case series have consecutive inclusion of participants?	Yes	Yes	Yes	Yes	Yes	Yes	4 Were participants blind to treatment assignment?	Yes
5 Did the case series have complete inclusion of participants?	Yes	Yes	Yes	Yes	Yes	Yes	5 Were those delivering the treatment blind to treatment assignment?	Yes
6 Was there clear reporting of the demographics of the participants in the study?	Yes	Yes	Yes	Unclear	Yes	Yes	6 Were treatment groups treated identically other than the intervention of interest?	Yes
7 Was there clear reporting of clinical information of the participants?	Unclear	Yes	Yes	Unclear	Yes	Yes	7 Were outcome assessors blind to treatment assignment?	Yes
8 Were the outcomes or follow up results of cases clearly reported?	Yes	Yes	Yes	Yes	Yes	Yes	8 Were outcomes measured in the same way for treatment groups?	Yes
9 Was there clear reporting of the presenting site(s)/clinic(s) demographic information?	Unclear	Yes	Yes	Unclear	Yes	Yes	9 Were outcomes measured in a reliable way?	Unclear
10 Was statistical analysis appropriate?	Yes	Yes	Yes	Yes	Yes	Yes	10 Was follow up complete and if not, were differences between groups in terms of their follow up adequately described and analysed?	Yes
Total score	7/10	9/10	9/10	7/10	10/10	8/10	11 Were participants analysed in the groups to which they were randomized?	Unclear
							12 Was appropriate statistical analysis used?	Yes
							13 Was the trial design appropriate and any deviations from the standard RCT design accounted for in the conduct and analysis of the trial?	Yes
							Total Score	11/13

JBI appraisal checklist for case reports	Madden and Bruera Madden and Bruera [48]	Weidner and Plantz Weidner and Plantz [49]
Were patient's demographic characteristics clearly described?	Yes	Yes
2 Was the patient's history clearly described and presented as a timeline?	Unclear	Unclear
3 Was the current clinical condition of the patient on presentation clearly described?	Yes	Yes
4 Were diagnostic tests or assessment methods and the results clearly described?	Yes	No
5 Was the intervention(s) or treatment procedure(s) clearly described?	Yes	Unclear
6 Was the post‐intervention clinical condition clearly described?	Yes	Yes
7 Were adverse events (harms) or unanticipated events identified and described?	Yes	Unclear
8 Does the case report provide takeaway lessons?	Yes	Yes
Total score	7/8	4/8

### 2.3 Data Analysis

Data were analysed using inductive content analysis, which is applicable to both qualitative and quantitative data [50]. From each selected study, general information—such as names of the authors, year of publication, study aim, design, setting, participants, data collection and data analysis method—was gathered and tabulated. The included articles were read thoroughly several times to gain a comprehensive understanding of the data. The results were then screened and relevant descriptions related to the research questions were extracted [31]. The analysis of the review was guided by the research questions.

For the first research question, the results were classified and tabulated based on the opioids used, adverse effects, patterns of opioid use and routes of administration [51]. The opioids used for pain management in the studies were categorized into potent opioids and weak opioids [52]. In the second research question, the units of meaning were expressions that described the family members' experiences of using opioids to treat a child's pain. A total of 13 original expressions were identified from the studies for analysis. These original expressions were simplified and grouped according to their similarities, leading to the formation of five subcategories. The synthesis of these subcategories led to three main categories: parental concerns, parental refusal and parental satisfaction [50]. The analysis process was conducted by the first author and then discussed and verified by the research group.

## 3 Results

### 3.1 Description of the Studies

In total, 16 research articles were included in this review. These articles were published between 1996 and 2022, with most being published after 2009. The studies were conducted in eleven different countries: the United States (*n* = 5), Australia (*n* = 2), Germany (*n* = 1), Egypt (*n* = 1), Wales (*n* = 1), England (*n* = 1), Finland (*n* = 1), China (*n* = 1), Canada (*n* = 1), Italy (*n* = 1) and Poland (*n* = 1). These studies were conducted in five different settings, which were cross‐sectional (*n* = 4), quasi‐experimental (*n* = 3), case report (*n* = 2), case series (*n* = 6) and randomized controlled trial (*n* = 1). In all studies, the child received care within a hospital setting, and in eight studies the child also received care in a home care setting (Table 2).

**TABLE 2:** Description of the studies reviewed.

Author, year, and country	Aim	Design and setting	Participants	Data collection	Data analysis
Anghelescu et al. [44], USA	Safety of epidural analgesia supplemented with intravenous opioids	Case series, hospital setting	Paediatric oncology patients receiving postoperative epidural analgesia (*n* = 117)	Patient demographics, details of pharmacological treatments, physiological measurements, and pain assessment results (scales used: numeric, FACES, FLACC) from medical records	Descriptive statistics, thematic content analysis
Collins et al. [47], USA	Safety and efficacy of an administering protocol, compare morphine and hydromorphone side effect profile, potency, and efficacy	Randomized controlled trial, hospital setting	Paediatric oncology patients having mucositis pain after bone marrow transplantation (*n* = 10)	Patient demographics and vital signs from medical records. Medication concentrations from blood specimens, nurses' assessments of sedation symptoms by using sedation symptom assessment scale, and patient‐rated pain assessments (scale used: VAS)	Statistical comparisons, crossover and between‐group comparisons, analysis of variance, and Friedman two‐way analysis of variance
Coombes et al. [41], England	Correlation between the effective dose of fentanyl and background dose of oral morphine	Retrospective cross‐sectional study, hospital setting	Paediatric oncology patients (*n* = 26)	Patient demographics, details of used opioid and other pharmacological treatments collected from medical records	Descriptive statistics, Spearman's rank correlation coefficient
Davies et al. [42], Canada	Safety and efficacy of methadone	Case series, hospital and homecare setting	Paediatric patients having advanced cancer related pain (*n* = 17)	Patient demographics, pharmacological treatment details and pain assessments from medical records	Descriptive statistics
Drake et al. [35], Australia	Therapeutic value of opioid rotation	Retrospective cross‐sectional study, hospital setting	Paediatric oncology patients on opioid therapy (*n* = 22)	Patient demographics, details of used opioid, adverse effect descriptions and pain assessments (Scales used: FACES, VAS) from medical records	Descriptive statistics, paired *t*‐test
Kubica‐Cielińska et al. [34], Poland	Morphine and nalbuphine side effect and therapeutic efficacy comparison	Cross‐sectional study, hospital setting	Paediatric oncology patients in primary disease treatment (*n* = 96)	Patient demographics, details of medications used, and pain assessments (Scales used: VAS, FACES, FLACC) from medical records	Descriptive statistics, Mann–Whitney *U* test, Shapiro–Wilk test, Chi‐Squared test, Friedman test, Kruskal‐Wallis test, and Spearman rank–order correlation analysis
Madden and Bruera [48], USA	Very‐low‐dose methadone as an effective strategy to treat refractory neuropathic pain	Case report, hospital and homecare setting	Paediatric oncology patients with refractory neuropathic pain (*n* = 2)	Patient demographics and details of medications used from medical records. Symptom severity by using parent/caregiver symptom assessment, and pain assessment (scale used: FACES)	Descriptive statistics
Madden et al. [46], USA	Efficacy and safety of methadone	Case series, hospital and homecare setting	Paediatric oncology patients with advanced cancer (*n* = 52)	Patient demographics, clinical information, and details of medications used from medical records. Symptom descriptions from assessments answered by parents or child (scale used: PSAS)	Descriptive statistics, Clopper–Pearson method, Wilcoxon signed‐rank test, Bonferroni correction and Kappa coefficient
Mashayekhi et al. [39], Wales	Characterize morphine and morphine 6‐glucurronide pharmacokinetics and pharmacodynamics	Quasi‐experimental study, hospital setting	Paediatric oncology patients (*n* = 7)	Patient demographics, details of medications used, and reported adverse effects from medical records. Medication concentrations from blood specimens and patient rated pain assessments (scales used: numeric, FACES).	Descriptive statistics, one‐compartmental model
Noyes and Irving [43], Australia	The usefulness of transdermal fentanyl	Case series, hospital and homecare setting	Paediatric oncology patients in palliative care (*n* = 13)	Patient demographics and pharmacological treatment details from medical records. Medication efficacy reported by nurse or parent (scale used: three‐step self‐made scale). Pain assessment by the child or parent	Descriptive statistics
Othman et al. [40], Egypt	Safety, efficacy and tolerability of transdermal fentanyl	Quasi‐experimental study, hospital and homecare setting	Paediatric cancer patients having chronic from moderate to severe cancer‐related pain (*n* = 64)	Patient demographics, pharmacological treatment details, physiological measurements, and sedation level measurements from medical records. Physical examination performed by physician. Child's assessment of pain (scales used: VAS, FACES) and adult's assessment of the child's level of play and activity (scale used: PSS)	Descriptive statistics, Mann–Whitney test, chi‐square test
Ruggiero et al. [38], Italy	Transdermal buprenorphine compliance, efficacy, and safety	Quasi‐experimental study, hospital and homecare setting	Paediatric patients with moderate to severe cancer related pain (*n* = 16)	Patient demographics and pharmacological treatment details from medical records. Assessment of pain (scale used: FACES), quality of sleep, the level of play and activity, alimentation, and speech, crying, treatment efficacy, and patient's compliance; self‐reported by school‐age children and reported by parents for younger children	Descriptive statistics, One‐way ANOVA, Bonferroni correction, Friedman non‐parametric test, chi‐square test
Schiessl et al. [45], Germany	Analyse the use of patient‐controlled analgesia	Case series, hospital and homecare setting	Paediatric oncology patients using intravenous patient‐controlled analgesia in palliative care (*n* = 8)	Patient demographics and pharmacological treatment details from medical records. Medication details from patient‐controlled analgesia pump's bolus‐and‐event recorders. Pain assessments reported by the child, parent, or health care professionals (scale used: numeric 0–10)	Descriptive statistics, Wilcoxon test
Sirkiä et al. [37], Finland	Adequacy or inadequacy of the analgesia achieved	Cross‐sectional study, hospital and homecare setting	Paediatric oncology patients in terminal care (*n* = 70)	Patient demographics, pharmacological treatment details, and length of terminal care from medical records. Structured parent interviews about child's disease, terminal care, and death	Descriptive statistics, retrospective analysis, inductive content analysis
Weidner and Plantz [49], USA	Recognizing the barriers to opioid use	Case report, hospital setting	Paediatric cancer patient having pain (*n* = 1)	Patient demographics from medical records, nursing staffs' reports about patient's appearance and pain occurrence, and the child's and parents' expressions to nursing staff	Review of descriptions
Yang et al. [36], China	Pain management in newly diagnosed sarcoma patients	Cross‐sectional study, hospital setting	Paediatric newly diagnosed sarcoma patients (*n* = 188)	Patient demographics, pharmacological treatment details, and adverse effect symptoms from medical records. Pain assessments systematically when administering analgesics (scales used: FLACC, FACES)	Descriptive statistics

Abbreviations: FACES = Wong Baker Faces scale, FLACC = Face, Legs, Activity, Cry, and Consolability, PSAS = paediatric symptom assessment system, PSS = the play and performance scale, VAS = Visual Analogue Scale.

In the studies included in this review, the age of the participating children varied between 3 months and 18.5 years (mean 9 years). In many of the studies, the mean age of the children ranged between 7 and 10.5 years. The phase of a child's cancer was described in 11 studies. In seven studies, the child was treated in palliative care [35, 37, 42, 43, 45, 46, 49], and in two studies the child was described as having advanced cancer [42, 46]. In one study the participants were newly diagnosed [36], and in one study the children were in remission and in maintenance chemotherapy [48]. In one study the participating children were undergoing primary disease treatment [34]. In five of the included studies, the phase of the disease was not described.

The sample size in cross‐sectional studies varied from 22 to 188 and from 7 to 64 in quasi‐experimental studies. In case reports, the sample sizes were one and two, and the sample size varied from eight to 117 in case series. In the randomized controlled trial, the sample size was ten. Most of the data in the studies were collected from medical records and assessment scales. In six studies, symptom assessment scales were used for data collection. In 10 studies, the use of standardized pain or symptom assessment scales was reported. The pain assessment scales used in the studies were the Wong–Baker Faces Pain Rating Scale [34, 35, 36, 38, 39, 40, 44, 48], the Face, Legs, Activity, Cry, Consolability scale [34, 36, 44], and the Visual Analogue Scale [34, 35, 40, 47]. Other standardized assessment scales used in the studies were the Paediatric Symptom Assessment System scale [46] and the Play and Performance Scale [40].

### 3.2 Opioid Use in Children

#### 3.2.1 Opioid Use

In total, the use of 11 different opioids was described. The most frequently used opioids for the treatment of pain in paediatric cancer patients were those classified as potent opioids. The most frequently used potent opioids in children were morphine [34, 35, 36, 37, 39, 40, 41, 42, 43, 44, 45, 47] and fentanyl [35, 40, 41, 42, 43, 44]. Other potent opioids used in children were hydromorphone [35, 42, 44, 46, 47], methadone [37, 41, 42, 46, 48] and oxycodone [41, 46]. The opioids least frequently used in pain management included nalbuphine [34], hydrocodone [46], tramadol [45] and codeine [46] (Table 3).

**TABLE 3:** Opioids used in children.

Opioid	Study source
**Potent opioids**	
Morphine	[34, 35, 36, 37, 39, 40, 41, 42, 43, 44, 45, 47]
Fentanyl	[35, 40, 41, 42, 43, 44]
Hydromorphone	[35, 42, 44, 46, 47]
Methadone	[37, 41, 42, 46, 48]
Oxycodone	[41, 46]
Buprenorphine	[37, 38]
Nalbuphine	[34]
**Weak opioids**	
Pethidine	[35, 37]
Tramadol	[45]
Hydrocodone	[46]
Codeine	[46]

The use of weak opioids was primarily delineated in studies published between 1998 and 2008 [35, 37, 45], whereas the use of morphine in pain management was highlighted in studies published between 2016 and 2022 [34, 36, 40, 41]. The use of fentanyl in pain management emerged through studies published between 2008 and 2017 [40, 41, 42, 44]. The use of nalbuphine was recently reported in an individual study [34].

The most common indication for opioid use in pain management was described as cancer pain [35, 36, 37, 38, 40, 41, 42, 43, 45, 46, 49]. Other indications for opioid use were pain caused by mucositis [34, 35, 47], postoperative pain [35, 44], breakthrough pain [38, 41], neuropathic pain [35, 48], bone pain and visceral pain [35]. A total of seven different pain assessment methods were used across the studies. The most commonly used tools were the FACES scale [34, 35, 36, 38, 39, 40, 44, 48] and the Visual Analogue Scale (VAS) [34, 35, 40, 47]. Several studies also reported the use of the FLACC scale [34, 36, 44] and the numeric rating scale [39, 44, 45].

The opioids were most frequently administered intravenously [34, 37, 39, 40, 41, 42, 44, 45] or enterally [36, 37, 39, 42, 43, 46, 48]. In addition, transdermal [38, 40, 41, 43] and subcutaneous routes of opioid administration were used [37, 41, 43]. Other routes of opioid administration were epidural [37, 44], sublingual [37, 41], intramuscular [37], intranasal [41] and lozenge [41]. Patient‐controlled analgesia pumps were also used for administering opioids and managing pain [44, 45, 47].

The average duration of opioid use in children ranged from 7 days to 13 months. The majority of regular opioid use durations ranged from seven days to 36 days [34, 36, 37, 42, 43, 45, 47]. The longer durations of opioid use were 58 and 66 days [37], and 13 months [48].

#### 3.2.2 Adverse Effects of Opioids

The most common adverse effects in children who received opioids were nausea [34, 35, 36, 38, 39, 41, 45, 47], drowsiness [34, 35, 39, 41, 42, 43, 47], vomiting [34, 35, 36, 38, 39, 45, 47] and itching [34, 35, 39, 40, 45, 47]. In individual studies, patch‐related skin irritation [43], urinary retention [45], hallucinations [42] and withdrawal syndrome [34] were also reported (Table 4).

**TABLE 4:** Reported adverse effects.

Adverse effects	Study source
Nausea	[34, 35, 36, 38, 39, 41, 45, 47]
Drowsiness	[34, 35, 39, 41, 42, 43, 47]
Vomiting	[34, 35, 36, 38, 39, 45, 47]
Itching	[34, 35, 39, 40, 45, 47]
Constipation	[34, 38, 39, 43, 45]
Mild respiratory depression	[36, 40, 42, 44]
Rash	[35, 40]
Patch‐related skin irritation	[43]
Urinary retention	[45]
Hallucination	[42]
Withdrawal syndrome	[34]

### 3.3 The Experiences of Family Members

No findings on the experiences of siblings of paediatric cancer patients were found. However, research on parental experiences was found in five studies [36, 42, 43, 48, 49]. Parents' experiences of opioids used for the pain management of a child with cancer were described through the following categories: parents' concerns, parents' refusal and parents' satisfaction.

The concerns of parents were described as concerns regarding the replacement of a pain patch, addiction and the sedative effect of opioids. Parents found it upsetting to replace pain patches when their child cried due to the discomfort experienced at the skin site [43]. Moreover, parents were also concerned about opioid‐related addictions [49], and they reported having negative connotations related to addiction treatment with the opioid [42]. Parents were also concerned about the sedative effect of opioids. In particular, they were worried that opioid use would decrease their child's ability to interact with the family [49]. Furthermore, results related to parental concerns about opioid use were reported, particularly in situations where the child was in palliative care [42, 43, 49].

Parents' refusal was described as a resistance to provide an opioid analgesia [42, 49], and resistance to provide an opioid even if it was recommended for the child [36]. Parents were also portrayed as believing that their child did not need opioids, even when the child was experiencing severe pain [36, 49]. Research findings related to both parental concerns and the refusal of opioid use emerged in studies where the child's pain was defined as cancer pain [36, 42, 43, 49].

Parental satisfaction with opioid use in the management of their child's pain was described as satisfaction with pain relief, improvement in quality of life and less stressful stay at home [43]. Parents also noted an improvement in their child's quality of life and found it positive that their child was able to tolerate a long car trip after receiving opioids for pain management [43, 48]. Parents also delineated improvements in their child's pain management after receiving an opioid [42].

## 4 Discussion

The aim of this review was to analyse the use of opioids in children with cancer and the experiences of family members regarding the use of opioids in pain management. These results show that a wide range of opioids with different routes of administration are used to treat pain in children with cancer. Limited research has, however, been conducted on the experiences of family members in the context of opioids used for pain management in children with cancer, with a notable absence of studies describing the experiences of siblings from this perspective. Parental experiences were particularly described as concerns and as a desire to refuse opioid use, although satisfaction with opioid use was also described.

In this review, analgesics classified as potent opioids were the most reported opioids used for pain management in paediatric cancer patients, potentially reflecting the heightened intensity of pain experienced by children with cancer. As has been shown in previous studies, pain emerges as one of the most substantial and formidable symptoms in paediatric cancer patients, persisting for a considerable duration throughout cancer treatment [7, 8, 29]. The results of this review are also consistent with prior research findings, highlighting morphine as the most common opioid used for pain management in paediatric cancer patients [18].

The largest number of research articles found in this review were on morphine use. It is still not straightforward, however, to conclude that morphine is the most used opioid in children with cancer. Indeed, consideration must also be given to those factors that might affect the prevalence of morphine in the research literature. For example, morphine is one of the oldest drugs in the world [53], which may affect the prevalence of the reporting of this opioid in some studies. Furthermore, as the oldest opioid, morphine may also be often compared to other opioids, emphasizing its prevalence in research articles. Research may also have focused on those hospitals where morphine is used the most. Therefore, for the comparability of the results of this review, it is important that the geographical distribution of the included research studies is noted, as the results are limited to research conducted in industrialized countries. In this review, opioids were also categorized into two groups: weak and potent. However, this classification is not entirely straightforward due to the existence of divergent viewpoints on the matter, particularly regarding the benefits of classification, as well as the various criteria used to classify an opioid as either weak or strong [54].

The results of this review show cancer pain, mucositis and procedure‐related pain as common indications for opioid use. However, the results also highlighted that children receiving opioids for cancer pain were often treated in palliative care. Previous research findings have also shown that surgical and cancer pain [9] as well as mucositis [10] are pivotal factors leading to opioid use in children with cancer. Several studies included in the review reported the use of similar pain assessment methods and tools in children and adolescents. A key consideration in paediatric pain assessment is the child's age and their ability to express their pain experience. Moreover, comprehensive assessment should encompass an evaluation of overall well‐being and behaviour, as these may offer observable signs of pain that the child is unable to recognize, articulate or communicate [14, 16, 17].

The adverse effects of opioid use in children identified in this review align with earlier research findings on the adverse effects of opioid use experienced by operative paediatric patients who potentially experience a distinct type of pain [19]. When considering the adverse effects of opioids experienced by a child with cancer, however, it should be noted that similar symptoms are also associated with the disease itself and with chemotherapy [4].

Paediatric oncology patients typically need the implementation of a central venous catheter or an alternative intravenous route for cytostatic treatment [3]. In this review, the intravenous route stood out as a common route for opioid administration in pain management in children. The common use of the intravenous route may be influenced by the presence of a catheter already used for chemotherapy. Furthermore, adverse effects experienced by the child, such as nausea and a loss of appetite, may also affect the child's ability to take medicines orally [4], thereby necessitating a different route of administration.

The findings of this study align with prior research regarding parental concerns about the adverse and addictive effects of opioids [22]. The cultural context of opioid use and the availability of different opioids in different parts of the world are also worth considering, particularly as five of the studies in the review were conducted in the United States. Over the past 30 years, three opioid crises have been identified in the United States, each having a significant impact on people and their experiences [20]. These crisis‐related experiences and perceptions can significantly influence parents' fears and concerns about opioid use in their children. Conversely, the cost of morphine preparations has been found to vary widely between countries [21], which can also vicariously affect the prescribing and availability of opioids for pain management.

The use of opioids and parents' experiences related to opioids were emphasized in the palliative care of children with cancer in this review. It is worth noting that the objectives of palliative care are not to cure cancer, but to focus on enhancing the child's quality of life. In contrast, treatments administered before palliative care are typically curative but often have the drawback of compromising the child's overall quality of life [25]. To further research the experiences of family members regarding opioid use, it is essential to consider the specific stage of treatment within the study population. Furthermore, it should be noted that the baseline between a child undergoing palliative care and one undergoing active cancer treatment may vary significantly, even if both have been diagnosed with cancer.

As previous studies have indicated that parents, upon a child's cancer diagnosis, commonly perceive pain as one of the most oppressive factors and may also have misconceptions regarding pain management in children [6, 29], further research on this topic is needed. The siblings of children with cancer need special support [28]; therefore, a comprehensive study on the experiences of siblings concerning opioid use as part of their sibling's pain management is relevant.

### 4.1 Limitations

This review has certain limitations that should be considered when interpreting the findings. First, the data search was limited to studies written in English, Swedish or Finnish, which may have affected the availability of relevant studies for this review. Second, the screening of the studies through title and abstract was conducted by one author, leaving the possibility of some pertinent studies being excluded from the review.

## 5 Conclusions

The use of opioids holds particular significance in the management of pain among children with cancer. For this reason, the use of opioids in this patient group needs to be studied in a comprehensive way, and healthcare professionals must be adequately trained in these diverse approaches and perspectives. A significant part of parents' experiences points to parental concerns and denial of opioid use for the child. Parents should, therefore, be consistently provided with information and guidance concerning the treatment of their child's severe pain and the analgesics used for its management. There is also a notable lack of research regarding the experiences of siblings in connection with the use of opioids for the management of a child's pain. This topic requires further research from these perspectives as well as on experiences during the different phases of cancer treatment. Methodologically, further research, particularly employing qualitative methods, is essential for enhancing family nursing.

## Author Contributions

**Noora Puusa:** conceptualization, methodology, investigation, formal analysis, writing – original draft and editing. **Eeva Harju:** conceptualization, methodology, investigation, formal analysis, writing – review and editing. **Maija‐Liisa Kalliomäki:** conceptualization, formal analysis, writing – review and editing. **Elina Haavisto:** conceptualization, methodology, investigation, formal analysis, writing – review and editing. All authors reviewed and approved the final version of the manuscript.

## Funding

The authors have nothing to report.

## Ethics Statement

The literature review is based on previously conducted studies; therefore, approval from an ethics committee was not necessary. Furthermore, no harm was caused to individuals as a result of the study, and ethical standards were adhered to throughout all stages of the research process.

## Conflicts of Interest

The authors declare no conflicts of interest.

## Data Availability

The data used in this study was collected from previously published scientific research articles.
